# Reconfiguring clinical communication in the electronic counselling context: The nuances of disruption

**DOI:** 10.1002/nop2.218

**Published:** 2018-11-20

**Authors:** Bjørg Oftedal, Beate‐Christin Hope Kolltveit, Marit Graue, Vibeke Zoffmann, Bjørg Karlsen, Sally Thorne, Margareth Kristoffersen

**Affiliations:** ^1^ Faculty of Health Sciences University of Stavanger Stavanger Norway; ^2^ Faculty of Health and Social Sciences, Centre for Evidence‐Based Practice Western Norway University of Applied Sciences Bergen Norway; ^3^ The Research Unit Women’s and Children’s Health The Juliane Marie Centre University Hospital Copenhagen Copenhagen Denmark; ^4^ School of Nursing University of British Columbia Vancouver BC Canada

## Abstract

**Aim:**

This study expands on an earlier study about diabetes nurses’ experiences of the Guided Self‐Determination intervention in face‐to‐face consultations among people with type 2 diabetes. This current study investigates Guided Self‐Determination in an electronic format with the aim to explore what can be learned about the written form for health communication from the perspectives of diabetes nurses in primary care.

**Design:**

The study has an explorative, qualitative design.

**Method:**

Four diabetes nurses were individually interviewed after completing the electronic intervention. The analysis was guided by Interpretive Description.

**Results:**

Small sample size apart, the rich empirical data and quality of dialogue point to the interviewees’ earlier contact, comfort and trust with the researcher. The written electronic communication could disrupt nurses’ possibilities for using basic and advanced communication skills. Findings also indicate that written electronic communication can foster thoughtful responses to patients and increase possibilities for a transparent counselling delivery process.

## 1 INTRODUCTION

The World Health Organization report on diabetes (WHO, 2016b) emphasises the enormous size and burden of diabetes worldwide. Globally, approximately 422 million adults aged over 18 years are living with type 2 diabetes (WHO, 2016b). The majority of those diagnosed with type 2 diabetes do not achieve the recommended glycaemic control, as it requires considerable self‐discipline and motivation concerning diet, exercise and blood glucose testing (WHO, 2016b). Inadequate blood glucose control may result in blindness, kidney failure, amputation and several other long‐term consequences that can significantly affect people’s quality of life (IDF, 2017). Consequently, people with type 2 diabetes need support delivered by skilled nurses to promote diabetes management and healthy choices (WHO, 2016b).

Electronic health technologies (eHealth) have been endorsed as tools for dealing with the rising numbers of people with type 2 diabetes. Today, electronic communication (eCommunication), particularly electronic mail (e‐mail) and text messages, has to some extent, supplemented face‐to‐face consultations among people with type 2 diabetes (Ye et al., 2010). However, technology itself will not transfer support and counselling of patients without healthcare professionals who have the knowledge and skills to effectively practise under this new digital paradigm of patient care. WHO (2016a) argues that the key for providing high‐quality eHealth services is training and continuing professional development of healthcare professionals. However, research addressing electronic communication between patients and nurses is in its early stages (Koivunen & Saranto, 2017).

This study is extending on an earlier published study about how diabetes nurses in primary care experienced the process of learning to practise the intervention Guided Self‐Determination in face‐to‐face consultations among people with type 2 diabetes (Oftedal, Kolltveit, Zoffmann, Hörnsten, & Graue, 2017). In this current study, we move from face‐to‐face consultation to Guided Self‐Determination in an electronic format with the purpose of developing knowledge about what may be needed to support nurses in shifting health intervention communications into the electronic format.

## 2 BACKGROUND

The advances in technology and the potential benefits for health care have resulted in a variety of eHealth interventions in clinical practice. Most of these interventions refer to medical and health support that is personalized, interactive and based on data obtained from patients, in contrast to information that patients may get from generalized websites on health and disease (Elbert et al., 2014). Many eHealth interventions are based on asynchronous communication (refers to communication in delayed time such as e‐mail and blogs), to foster communication whenever it is convenient for the healthcare professionals or patients, thus freeing the patient from the necessity of travelling to the clinic at a precise time to receive counselling (Huxley, Atherton, Watkins, & Griffiths, 2015). A systematic review (Ye et al., 2010) shows that the use of e‐mail in patient–provider communication has the potential to improve health communication between patients and providers, thus increasing satisfaction and the quality of care. Other studies reveal that electronic communication between healthcare professionals and patients can improve patient‐centred care (Cornwall, Moore, & Plant, 2008), facilitate patient information (Kinnane, Milne, Kinnane, & Milne, 2010), prevent dropout and deterioration of patients’ health (Das, Faxvaag, & Svanæs, 2015). However, eHealth interventions for people with type 2 diabetes appear to vary widely in terms of follow‐up, length and quality (Pal et al., 2013). Previous systematic review reveals the need for eHealth interventions based on a theoretical framework that would provide appropriate patient‐tailored feedback from healthcare professionals (Pal et al., 2013). A recent study, using a theory‐driven intervention, shows that diabetes nurses (DNs) experienced this Guided Self‐Determination (GSD) intervention for type 2 diabetes as a constructive counselling method for stimulating patients’ reflections and motivation for diabetes management (Oftedal et al., 2017). Based on these findings, we, in this current study, adapted the GSD approach to an asynchronous eHealth intervention based on written communication. However, incorporating eHealth in clinical practice may represent a disruptive change in the health care and work settings (Koivunen, Niemi, & Hupli, 2015) and a review has identified these challenges; lack of knowledge about the eHealth technology, perception of usefulness, lack of time, training or limited access (Ye et al., 2010). It is argued that if healthcare professionals are dissatisfied with the eHealth intervention, it is unlikely that an intervention would be implemented (Li, Talaei‐Khoei, Seale, Ray, & Macintyre, 2013). Therefore, an effort should be made to investigate what is actually taking place when eHealth intervention is implemented in clinical practice. More specifically, it is important to investigate the unintended consequences of eCommunication among nurses in clinical setting (Melby & Hellesø, 2014). A systematic review stated that eCommunication between patients and nurses has not really been studied from the nursing professionals’ point of view (Koivunen & Saranto, 2017). In addition, no studies have explored nurses’ perspectives on the use of GSD intervention in written eCommunication portals geared towards people with type 2 diabetes in primary care.

### 2.1 Aim

The aim was to explore what can be learned about the written form for health communication from the experiences of diabetes nurses using an asynchronous electronic Guided Self‐Determination intervention for people with type 2 diabetes in primary care. Towards this end, the study was guided by the following question: From a nursing perspective, what is gained and what is lost through written electronic communication?

### 2.2 Design/Method

This small exploratory study has a qualitative design with an Interpretive Description (ID) methodology (Thorne, 2016), which is a qualitative research strategy developed for the purpose of advancing knowledge in the applied disciplines. As ID is particularly relevant to questions arising from daily clinical practice and for developing new knowledge and insight about clinically topic that may generate changes in health care, the methodology was ideally suited to this study. The data were collected in 2016 by means of individual interviews with four DNs in general practice, representing rural and urban municipalities in south‐western Norway.

### 2.3 Electronic Guided Self‐Determination intervention

In this study, the eHealth intervention was based on GSD developed for people with type 2 diabetes (Karlsen et al., 2016). The GSD is a theory‐driven counselling approach founded in the synthesis of grounded theories, self‐determination theory, life skills theory and humanistic values theory (Zoffmann et al., 2016; Zoffmann, Harder, & Kirkevold, 2008). The purpose is to guide patients and health professionals through mutual reflection (Zoffmann & Lauritzen, 2006) using a six‐stage interaction process: (a) establishment of a mutual person–nurse relationship with clear I‐you‐borders; (b) self‐exploration; (c) self‐understanding; (d) shared decision‐making; (e) action; and (f) feedback from action. This process is facilitated by the use of several semistructured reflection sheets that are intended to empower the patient to become self‐determined and to develop adequate life skills for managing challenges in diabetes management. Four themes are included in the reflection sheets: the patient–provider relationship, life with diabetes, the relationship between the ideal and reality and working to change. To encourage patients’ capacities to become active and autonomous problem‐solvers, the nurses rely on three advanced communication skills: active listening, mirroring and values‐clarifying responses. The intervention consisted of four eConsultations (Table 1) which were conducted over a period of 12–35 weeks. Initially, the DN and the patient with type 2 diabetes met face‐to‐face to establish a relationship and to clarify the aim of the counselling. In addition, all patients received a manual for the web page. The manual provided instructions on how to download the PDF files, write in the reflection sheets and send secure messages. The secure messaging system was provided by the portal www.MinJournal.no. (“My Chart”), developed at the Oslo University Hospital, Norway. The portal requires login with an electronic identification (BankID), which is required by Norwegian law for transferring sensitive information online. After the initial face‐to‐face meeting with patients, four eConsultations were conducted. The patients completed reflection sheets following the eConsultations and sent these to their DN via secure messages. The DNs responded asynchronously in writing to the patients’ reflections using the advanced communication skills mentioned above. Each of the four eConsultations involved exchanges of 2–4 electronic text messages.

**Table 1:** eGSD intervention

The first session at the GPs office	Preparing for subsequent consultations: Invitation to work together The HbA1c measurement
eConsultations and focus:	Reflection sheets (RS)
(1) Your life with diabetes	Important events and periods in your life At present, what do you find difficult about living with diabetes? Unfinished sentences—your needs, values, habits and opportunities A picture, metaphor or expression of your life with diabetes
(2) Focus for change	Room for diabetes in your life Your plans for changing your way of life
(3) Work with changes	Clarification of challenge in your life with diabetes Previous problem‐ solving: thoughts, feelings, goals and actions Dynamic problem‐solving
(4) Changes in daily life	Blood glucose self‐monitoring and your reasons for self‐monitoring New strategies and long‐ term plan for change Dynamic judgement of current and future problem‐solving

### 2.4 Sample

A purposive sample of diabetes DNs was selected from general practitioners (GPs). However, potential recruits for this study were relatively few, as not many GPs in Norway employ Registered Nurses. Therefore, to obtain as many recruits as possible, the first author (BO) disseminated information about the study during a professional meeting for DNs and by telephone to GPs in south‐western Norway, inviting them to participate. The inclusion criteria for nurses were as follows: Nurses must: (a) be Registered Nurses and employed in a general practice; (b) have more than 1 year of experience in diabetes care; (c) have completed a test about the GSD; and (d) be willing to use GSD in an electronic written format. The head of the practice approved the participation of the nurses in the study. Five Registered Nurses with particular diabetes expertise were invited to participate. One DN was reported sick. In total, four DNs consented to participate. All study participants were women, aged from 36 ‐ 56 years. The median duration of their experience in diabetes care was 7 years (ranging from 4 ‐ 22 years). Three of the four DNs had formal postgraduate education in diabetes care (60 ECTS) and all had been trained in the GSD method (Oftedal et al., 2017). However, none of the DNs had been trained in GSD on an eHealth platform.

### 2.5 Data collection

Individual interviews were performed with the DNs after they had used the electronic GSD platform for patients with type 2 diabetes (spring 2016). The research BCHK, who is also a registered diabetes nurse, conducted the interviews and each interview took place at the University and was limited to 1 hr. A thematic semistructured interview guide was developed by the authors (BO & BCHK), including follow‐up questions designed to make it possible for the study participants to highlight perspectives relevant for the research topic. First, DNs were asked an initial question about their overall experience with the eGSD for patients with type 2 diabetes. This question was followed up by more specific questions concerning their experiences using written electronic communication with patients and how they experienced the patient relationship when counselling via Internet. The interviews were audiotaped and transcribed verbatim. The text was imported into QSR International’s NVivo 11™ software programme for analysis.

### 2.6 Data analysis

The analysis was guided by Interpretive Description (ID; Thorne, 2016). An important factor in ID is to begin with an open‐minded reading of the transcribed text to obtain the sense of the whole. Three members of the research team (BO, BK and MG) read the text several times, without focusing too much on details at this early stage of the analysis, but roughly coding the sequences that were considered important for the analysis. Questions asked of the material during this initial reading and re‐reading process were as follows: What is seen? What is going on and what does it mean? Following this first preliminary coding, a closer examination of the labelled statements was conducted and general questions such as what is the main message in the material and what new understanding can the data provide were asked of the data (Thorne, 2016). In this study, relevant analytic questions included: In what ways do the DNs describe and explain their experience with eGSD? What do they describe as lost through written electronic communication and why and what kinds of experiences have they gained through the written electronic communication and why? Through this analytic questioning process, the codes were initially grouped into tentative patterns. The research team discussed the patterns and the relationship between these patterns and sections in the material and concluded the analytic process by conceptualizing the findings in a form that illustrated the application of the GSD intervention based on written eCommunication from the perspective of these diabetes nurses.

### 2.7 Ethics

The Norwegian Social Sciences Data Services approved the study (No. 39454). All respondents provided informed written consent before the individual interviews and were guaranteed confidentiality and the right to withdraw from the study at any time. The anonymity of participants was maintained by removing names from records and transcriptions.

### 2.8 Findings

The main finding of this study was that nurses did find the written eCommunication to be a disruptive format in their capacity to deliver care. However, this disruption presented both advantages and disadvantages to the clinical context, which became apparent as they reflected on the gains and losses associated with this form of care delivery. Their interpretation of the nature of this disruption is presented here in the context of three major themes that emerged from their accounts, each of which reveals elements of the nuances of these changes. The following themes were identified: (a) Replacing basic and advanced communicative skills with written expressions; (b) Making process transparent; and (c) Creating space for reflection and insight. Quotations from the interviews are presented to illustrate how nurses expressed their experiences with the asynchronous GSD written eCommunication.

### 2.9 Replacing basic and advanced communicative skills with written expressions

The nurses said that eCommunication, as a result of its reliance on electronic, written function, changed their way of communicating with the patients and represented other aspects than in an oral dialogue. One aspect that emerged from the analysis was that nurses experienced the electronic, written communication with patients to be challenging because the various layers of communication that occur during face‐to‐face encounter were lost in an written eCommunication. For example, emotional cues from vocal intonation or body language were missing. In particular, nurses highlighted that body language such as eye contact, posture and gesture, and facial expressions and the general non‐verbal expression was lacking. These expressions helped nurses identify the reactions, questions and needs of patients, which then facilitated nurses’ understanding and informed their responses, often without words. They described the written eCommunication as “totally free of body language” due to the inability to read non‐verbal expression. In the absence of such expressions and interaction with patients, nurses lost the opportunity to better align their messages with patients need. For example, one nurse expressed the following:

I like eye contact and interpreting body language and moods. I cannot do that between the lines. When you speak to the patient, I can tell when they seem skeptical. This is not caught up online, because it is so filtered (3).

When communicating face‐to‐face, nurses said that they could use more of their senses to assess the patient’s expressions or voice and thus evaluate their emotional or mental state of the patient. On the other hand, the absence of non‐verbal communication was also perceived as an advantage. One nurse experienced face‐to face communication as noisy and as a barrier to the reflective responses to patient in face‐to‐face setting, stating, “You are affected by expression, sounds. All senses are fully operational” (3).

The use of advanced communication skills such as mirroring and active listening was reported as difficult under the scheme of eGSD counselling. Some nurses reported experimenting with different text expressions such as ellipses (….), question marks, cursive or boldfaced type and used of emojis when trying to mirror interactions with the patient. However, despite the use of creative writing, they found it difficult to use the advanced communication skills they had learnt as part of the GSD training. One nurse reported the following: “I tried with value clarification responses to the degree it was possible to use these tools, but it wasn’t easy. I was aware that this was the GSD I would be able to offer” (4). Another aspect that characterized the nurses’ experience of written eCommunication was how it changed their relationship with the patients. Nurses described the relationship with patients to be good and constructive, but also depicted is as being more distant. As a result, closeness to the patient was lost as expressed by one nurse:

“The relationship was good, but it was distanced. I felt like they (the patients) were out in the world and I was here. I felt like I was missing something—a sense of closeness both parties needed” (3). Similarly, the asynchronous environment reduced nurses’ feelings of “being there” with the patient, which could be potentially detrimental to building a therapeutic relationship. In this case, face‐to‐face contact offers richer stimuli, including auditory, visual, tactile and behavioural stimuli and smells and gestures, which were reported as being lost in the electronic environment with patients. On the other hand, nurses also reflected that written eCommunication with patients could sometimes be perceived as personal as expressed by one nurse:

…I think that when it was in writing, it became personal. Here’s someone who’s at home and writing to me via e‐mail or in my chart and responded with «Hi Peter», or whatever their name was. Kind regards, xx, turned into a friendly but not completely informal response, so I still think they felt considered (4).

### 2.10 Making process transparent

This theme reflects on how using electronic written, instead of verbal communication, makes the counselling process transparent. The nurses reported that the written, electronic environment provided an easier means for following the patient’s progress during the GSD, as the written text could be read repeatedly by both patients and nurses. Thus, this transparency extended in both directions. Some nurses noted that it could increase their ability to provide appropriate counselling and support. However, the analysis also revealed that other nurses perceived this process to be challenging, as text is irrevocable and they therefore lost the possibilities to change the text. They described certain difficulties in writing and reported that they often wrote, deleted and rewrote messages while reflecting on how patients would perceive the message. One nurse said the following: “At many points I would reconsider the responses I gave, but when you’re talking with someone across from you, you don’t spend nearly as much time considering what you should answer as you do when it is in writing” (2).

The nurses also stressed that written eCommunication is not a neutral tool and carries the risk of content being misinterpreted. They were aware that text could be perceived as much harsher or more powerful than verbal communication. As a consequence, the nurses spent a lot of time constructing sentences and formulating the answer to reduce the risk or avoid misunderstandings that potentially could harm a constructive relationship with patients. Some nurses reported performance anxiety in regard to written eCommunication because they were afraid that their written text was not good enough. One nurse reported the following:

I’ve found it difficult. It’s a new way to do it. I spent a lot of time considering what and how I should write: How will she interpret this – and feeling a certain performance anxiety about what is written in black and white – is it good enough? (1)

However, some nurses reported that practicing communication in writing also led to awakening and better communication with the patients in face‐to‐face consultations.

### 2.11 Creating space for reflection and insight

This theme highlights how the nurses perceived written eCommunication to be a tool for reflection, prompting them to take time in composing messages and reflecting on them before sending them to patients. Compared with face‐to‐face consultations, which require an immediate response to the patient, nurses found written eCommunication to result in messages that were well thought out and that was reflected on more deeply before responding to patients. For example, one nurse expressed the following: “It makes you more conscious about writing words to send. In that way I spent more time thinking about what I respond to the patient and what you should ask about and what you write” (2). In addition, some nurses appreciated that asynchronous eCommunication made it possible to read patients’ narratives when they were alone and not in face‐to‐face consultations with patients. This point of view was related to the fact that some patients’ narratives affected them strongly and, therefore, they appreciated having time alone to better apprehend these narratives. In this sense, nurses observed that some patients found it easier to share their challenges with diabetes in written form rather than during verbal consultation with the nurses. These written narratives from patients gave nurses a deeper insight into patients’ thoughts and experiences as expressed by the following quotation:

But at the same time, with the patient I’m working with now, she writes a lot. She has a lot of thoughts that haven’t been present in the yearly checkups. She writes a lot about herself and her own and her family’s background and why she feels this way. A lot of it has come out when she sat down to write (1).

The nurses reported that the eGSD approach had reoriented their support from giving diabetes advice and information to prompting patients’ responsibility for their own health. They also stated that they spent a lot of time to reflect on how to stimulate patients’ reflections, decision‐making and choice in an electronic written format as expressed by one nurse: “I will constantly write what I think the patient should do”(2). Apparently, writing requires more attention, reflection and time than verbal expression. In this respect, all nurses reported that the written eCommunication was time‐consuming. However, they also found time spent to be a constructive aspect. For instance, since eCommunication takes advantage of the delivery of asynchronous messages, nurses had time to obtain the information needed to respond to patients, including information they did have immediately at hand or in mind. Another aspect the nurses highlighted as an advantage was that asynchronous written eCommunication offers flexibility in regard to responding to patients. Nurses could thus respond to patients when they had time and without additional influencing factors. For example, one nurse reported the following:

The thing that was positive about it all was when I got the reflection sheets back from the patient. I could sit down in peace and quiet and read through without being disturbed by patients or others. What does it say between the lines? What does it really say? From that I could offer feedback. In one way it was easier when it was in digital formats than face to face (3).

On the other hand, they were concerned about how to maintain their professional role in written eCommunication. That was particularly expressed when they reflected over how asynchronous written eCommunication allowed them to be available 24/7 and thus responding to patients whenever they had time. Consequently, they were worried that responding to patients outside working hours could influence the nurses’ professional role in a negative way.

## 3 DISCUSSION

The aim of this study was to explore what can be learned about the written form for health communication from the experiences of diabetes nurses using an asynchronous electronic Guided Self‐Determination intervention for people with type 2 diabetes in primary care. The findings of this study challenge us to reflect on the potential and limitations of written electronic communication and to add to our understanding of what might be needed to support healthcare professionals in shifting health intervention communications into an electronic format.

### 3.1 Rethinking the essence of communication

The findings of the current study indicate that the asynchronous eGSD intervention requires new ways of communicating and a rethinking of the essence of communication between nurses and patients. Particularly, the findings show that a lack of non‐verbal communication cues became a important challenge for the nurses. A similar observation was also reported in another study investigating non‐verbal communication in text‐based medical consultation among physicians (Björk, Hillborg, Augutis, & Umefjord, 2017). Thus, this finding is not surprising, as non‐verbal communication is an inherent value for healthcare professionals to give comprehensive care to patients. Conversely, our study also indicates that the absence of non‐verbal communication can increase nurses’ reflective responses, as their interpretation was not affected by patients’ non‐verbal expression. Accordingly, in the light of our findings and assuming that use of digital written communication will continue to increase in clinical settings, it seems timely to question the degree to which the absence of non‐verbal communication contributes to or detracts from clinical practice. Alternatively, and more specifically, it will be important to gain an understanding of situations in clinical practice where non‐verbal communication becomes unnecessary to give patients adequate support. Is it possible that written communication in some situations could be more beneficial for both patients and nurses than face‐to‐face communication? Nurses and other healthcare professionals who are directly involved in the consequences of eHealth in clinical practice and the patients who are directly affected by these communications all have a key role to play in promoting the debate that will allow us to decide on best practices.

Another factor that was reported as lost when using asynchronous written eCommunication was the opportunity of using advanced communication skills like active listening or mirroring. Our study suggests that several creative writing strategies, such as the use of emoji, were developed by the nurses to compensate for advanced communication methods. Yet, the nurses reported they did not feel they succeeded with this strategy. This finding may indicate that written eCommunication is not necessarily a reduced form of face‐to‐face communication, but offers an entirely different vehicle for communication with its own unique advantages and limitations. Therefore, our findings support the findings of previous study reporting that there is a need to improve communication skills in written texts (Björk et al., 2017; van Houwelingen, Moerman, Ettema, Kort, & Cate, 2016) and that educational efforts to include written eCommunication in the nurse students curricula should be prioritized, as it is likely that text‐based consultations will expand in the future (Booth, 2006). Healthcare professionals and clinical educators could be a key conduit for stimulating change and increasing the focus on eCommunication.

### 3.2 Achieving a more transparent and reflective counselling

It is well known that electronic devices augment transparency and the findings of this current study support the position that written eCommunication may increase the opportunity of achieving a more transparent communication form in the counselling delivery process. Accordingly, the current findings become an important indicator that we will need to recognize and attend to electronic intervention as a factor contributing to a more transparent healthcare system. Indeed, the findings indicate that nurses support a culture that is open and transparent, as this makes it easier for them to follow patients during the counselling process. The WHO (2017) argues that the transparency of all communications is essential to building trust in health care. Silverman, Draper, and Kurtz (2016) also advocate that transparency promotes relationship building and reduces unnecessary patient uncertainty about their care. However, because transparency requires that a written text remains online, the findings indicate that this made the nurses in this study more cautious in formulating their feedback. To reduce the risk of misinterpretation, they spent much time preparing and formulating their texts to ensure that they were correct. They said that they were concerned that the patients would perceive the written texts as harsh and strict and they emphasized how difficult it was to lose the opportunity to change the texts and adjust them according to the patients’ immediate responses. Accordingly, in the light of our findings, when introducing eGSD to clinical practice, it is important to consider that asynchronous written eCommunication may be worrisome to some nurses who are concerned about the accuracy of written messages. It may also be suggested that not all nurses are sufficiently comfortable with posting written communications and there may need to be definable skill sets to allow nurses to practise in this manner. Nevertheless, the nurses’ concerns about how the patients would perceive their written communication are relevant, considering that several studies have revealed that many people cannot understand nor use written health information properly (Bailey et al., 2014; Jacobs, Lou, Ownby, & Caballero, 2016). It may, therefore, be assumed that written eCommunication could act as a reminder to help nurses become more conscious about the words and concepts they employ when consulting with patients. This interpretation is supported by a previous study (Björk et al., 2017) and our current study, which reveal that practicing written eCommunication can also lead to better communication skills in face‐to‐face consultations. This interesting perspective should be further investigated.

Another important finding was that the asynchronous environment made it possible to support the reflective responses that are valuable in GSD counselling, as nurses could read and respond to patients’ narratives when they were alone and not having face‐to‐face consultations with patients. Consequently, the feedback to patients was well thought out and deeply reflected on. Therefore, it seems possible that asynchronous eHealth intervention could help surmount the disruptive factors that are often encountered in face‐to‐face counselling. These findings are supported by other studies, which reveal that text‐based consultation gives healthcare professionals time to think, reflect and fine‐tune their answers (Björk et al., 2017; Dunn, 2012), which is a unique opportunity that is rarely available in face‐to‐face interactions.

### 3.3 Limitations

In this exploratory study, we acknowledge that the sample is small. Malterud, Siersma, and Guassora (2016) emphasize that a study with clear and focused dialogue between researcher and participants requires fewer participants to offer sufficient data material than a study with an unclear or vague communication. In our study, the researcher (BCHK) has interviewed the participants in an earlier study (Oftedal et al., 2017) and has thus, already established contact and trust in the dialogue. In addition, the researcher has knowledge of both the diabetic work at GP and the GSD intervention. We therefore consider the data material, consisting of rich and varied accounts, to be trustworthy. It permits a preliminary understanding that adds to our knowledge of what might be needed to support healthcare professionals while shifting health intervention communications into an electronic format. However, we could not preclude that a large number of participants might have thrown open different perspectives.

Another limitation is that the nurses in this study were born before 1980 and are, therefore, “digital immigrants” (Prensky, 2005). That means that although many aspects of the technology might be adopted, just like those who learn another language later in the life, they retain an “accent.” It is, therefore, possible that nurses born after 1980 might have identified other dimensions of asynchronous written communication. It is also unknown whether these findings related to eCommunication would have changed had the nurses used the written asynchronous communication over time.

## 4 CONCLUSION

The findings indicate that asynchronous written eCommunication could disrupt nurses’ possibilities to use basic and advanced communication skills and that written expression may not currently be adequate for replacing the communication skills that are traditionally used in clinical practice. However, the findings also suggest that asynchronous written eCommunication can foster deep and thoughtful responses to patients and as nurses become more conscious of the words they employ when responding in writing, they may enhance their communication skills in subsequent face‐to‐face interactions. In addition, written eCommunication increases the possibilities for intensified transparency of the counselling delivery process. Although much remains to be learned with regard to reconfiguring health intervention communications into electronic formats, this study highlights the potential advantages and limitations of using asynchronous written eCommunication in primary care.

## CONFLICT OF INTERESTS

We declare no conflict of interests.

## AUTHOR CONTRIBUTIONS

BO, MG, VZ and BK designed the study. BO and B‐CHK were involved in data collection. BO, MG, MK, ST, B‐CHK and BK analysed the data. BO has mainly drafted the manuscript, but all authors have contributed to the drafting of the manuscript, revised it critically for scientific content, read and approved the final version.

## ETHICAL APPROVAL

The Norwegian Social Sciences Data Services approved the study (No. 39454). All respondents provided informed written consent before the individual interviews and were guaranteed confidentiality and the right to withdraw from the study at any time.
